# Apigenin and hesperidin augment the toxic effect of doxorubicin against HepG2 cells

**DOI:** 10.1186/s40360-019-0301-2

**Published:** 2019-05-03

**Authors:** Agnieszka Korga, Marta Ostrowska, Aleksandra Jozefczyk, Magdalena Iwan, Rafal Wojcik, Grazyna Zgorka, Mariola Herbet, Gemma Gomez Vilarrubla, Jaroslaw Dudka

**Affiliations:** 1 Independent Medical Biology Unit, Medical University of Lublin, 8b Jaczewski Street, 20-090 Lublin, Poland; 2 Department of Toxicology, Medical University of Lublin, 8b Jaczewski Street, 20-090 Lublin, Poland; 3 Department of Pharmacognosy with Medicinal Plant Laboratory, Medical University of Lublin, 1 Chodzko Street, 20-093 Lublin, Poland; 4 Department of Human Anatomy, Medical University of Lublin, 4 Jaczewski Street, 20-090 Lublin, Poland

**Keywords:** Doxorubicin, Apigenin, Hesperidin, Flavonoids, Hepatocellular carcinoma

## Abstract

**Background:**

Hepatocellular carcinoma (HCC) is one of the most common malignancies, with an increasing incidence. Despite the fact that systematic chemotherapy with a doxorubicin provides only marginal improvements in survival of the HCC patients, the doxorubicin is being used in transarterial therapies or combined with the target drug – sorafenib. The aim of the study was to evaluate the effect of natural flavonoids on the cytotoxicity of the doxorubicin against human hepatocellular carcinoma cell line HepG2.

**Methods:**

The effect of apigenin and its glycosides - cosmosiin, rhoifolin; baicalein and its glycosides – baicalin as well as hesperetin and its glycosides – hesperidin on glycolytic genes expression of HepG2 cell line, morphology and cells’ viability at the presence of doxorubicin have been tested. In an attempt to elucidate the mechanism of observed results, the fluorogenic probe for reactive oxygen species (ROS), the DNA oxidative damage, the lipid peroxidation and the double strand breaks were evaluated. To assess impact on the glycolysis pathway, the mRNA expression for a hexokinase 2 (HK2) and a lactate dehydrogenase A (LDHA) enzymes were measured. The results were analysed statistically with the one-way analysis of variance (ANOVA) and post hoc multiple comparisons.

**Results:**

The apigenin and the hesperidin revealed the strongest effect on the toxicity of doxorubicin. Both flavonoids simultaneously changed the expression of the glycolytic pathway genes - *HK2* and *LDHA*, which play a key role in the Warburg effect. Although separate treatment with doxorubicin, apigenin and hesperidin led to a significant oxidative DNA damage and double strand breaks, simultaneous administration of doxorubicin and apigenin or hesperidin abolished these damage with the simultaneous increase in the doxorubicin toxicity.

**Conclusion:**

The obtained results indicate the existence of a very effective cytotoxic mechanism in the HepG2 cells of the combined effect of doxorubicin and apigenin (or hesperidin), not related to the oxidative stress. To explain this synergy mechanism, further research is needed, The observed intensification of the cytotoxic effect of doxorubicin by this flavonoids may be a promising direction of the research on the therapy of hepatocellular carcinoma, especially in a chemoembolization.

## Background

Doxorubicin (DOX), one of the most effective anticancer agents, has been widely used in anti-tumour Hepatocellular carcinoma (HCC) is one of the most common malignancies, with an increasing incidence [1, 2]. Despite the fact that the systematic chemotherapy with a doxorubicin provides only marginal improvements in a survival of the HCC patients, the doxorubicin is being used in transarterial therapies or combined with the target drug – sorafenib [3, 4]. The primary mechanism of action of the DOX involves the drug’s ability to intercalate within DNA base pairs causing a breakage of DNA strands and an inhibition of both DNA and RNA synthesis. The DOX inhibits the enzyme, topoisomerase II, causing the DNA damage and induction of apoptosis [5]. The second mechanism is connected with generation of the reactive oxygen species (ROS) by DOX which causes cell death in both cancer and normal cells. Formation of the ROS in cardiomyocytes, leading to fatal heart failure, is one of the most critical side effects of DOX treatment [6, 7].

In this work, we attempted to induce a pharmacological synergism of the DOX with compounds of natural origin in relation to HCC. In our preliminary studies, flavonoids showed inhibitory effects on the expression of hexokinase 2 (*HK2*) and lactate dehydrogenase A (*LDHA*) genes encoding key enzymes of the glycolytic pathway. A glycolysis activation is observed in many cancers and is accompanied by an increased tumour aggressiveness. Pivotal research in the 1920s by Warburg and Cori demonstrated that cancer avidly consumes glucose and excretes lactate [8, 9]. In some neoplasia the arising glucose consumption may be one order magnitude higher than in normal cells from which the neoplasia derives. Therefore, it was assumed that cancer cells generated energy using the glycolysis pathway rather than mitochondrial oxidative phosphorylation, and that the mitochondria were dysfunctional. The phenomenon of aerobic glycolysis, termed the Warburg effect, is not consistent across all cancer types [10], but about 80% of cancers demonstrate Warburg effect what is being used in PET diagnostics [11]. The studies on the effectiveness of glycolysis inhibition in the treatment of cancer seems justified and promising [12–15]. In addition, glycolysis inhibition combined with DNA damaging chemotherapeutic agents may be an effective anticancer strategy through weakening cell damage repair capacity and enhancing drug cytotoxicity [15].

The flavonoids (apigenin and hesperidin) have been shown to inhibit glycolysis thereby altering the metabolic phenotype. As a result, cancer cells may be less aggressive and more sensitive to therapy [16, 17]. Thus, flavonoids can sensitize cells to the DOX treatment. For this reason, the aim of the study was to evaluate the effect of natural flavonoids on the cytotoxicity of DOX against human hepatocellular carcinoma cell line.

## Methods

### Cell culturing and treatment

**Fig. 1:**
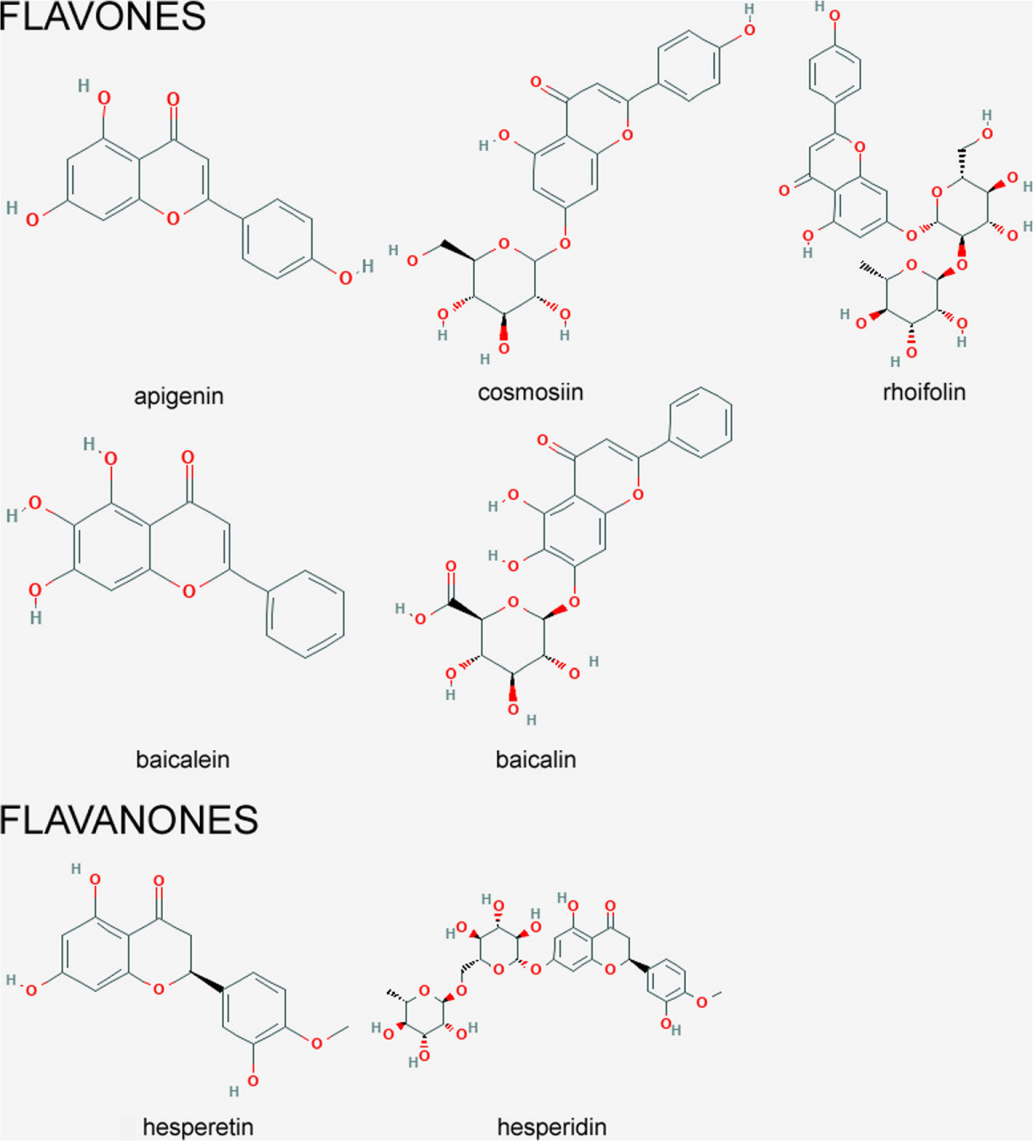
Chemical structures of tested flavonoids

The culture of human hepatocellular carcinoma (HepG2, HB-8065; ATCC, USA) was performed in Eagle’s Minimum Essential Medium (USA, ATCC) supplemented with 10% foetal bovine serum (Life Technologies, USA). Cells were incubated at 37 °C with 5% CO_2_ in air atmosphere. The tested cells were incubated for 24 h with 1 μM DOX (EBEWE Pharma, Unterach, Austria) and 25–200 μM of following HPLC standards (Sigma-Aldrich, USA): apigenin, cosmosiin, rhoifolin, baicalein, baicalin, hesperetin, hesperidin (Fig. 1) or combined (DOX + single HPLC standard). The tested concentration of DOX was based on reported clinically achievable plasma concentrations [18] and observed cytotoxicity for HepG2 cells.

### The cell morphology

The cell morphology was analysed under a phase-contrast microscope Nikon Eclipse Ti using NIS-Elements Imaging Software (Nikon, Tokyo, Japan).

### The cytotoxicity analyses

The cytotoxicity was evaluated using the MTT Assay Kit (Life Technologies, USA). The test is based on the living cells’ ability to reduce orange tetrazolium salt to water-insoluble purple formazan crystals. The cells were seeded into 96-well plates in the concentration of 1.5 × 10^5^ cells/mL. The tested compounds were added when 70–80% of confluence was achieved. The MTT solution (4.0 mg/mL) was added to the culture 24 h after chemicals. Following 4 h incubation, the medium with MTT was removed, and the formed crystals were dissolved in DMSO. The solution absorbency was measured at 540 nm, using PowerWave microplate spectrophotometer (BioTek Instruments, USA). Each assay was conducted three times and was measured in triplicates.

### Reactive oxygen species (ROS) detection

The CellROX Green Reagent (Invitrogen, USA) was used as the ROS indicator. The CellROX is a fluorogenic probe which is weakly fluorescent while in a reduced state. It exhibits bright green photostable fluorescence upon oxidation by ROS and subsequent binding to DNA, with absorption/emission maxima of 485/520 nm.

The cells were seeded into 6-well plates in concentration of 1.5 × 10^5^ cells/mL. The tested compounds were added when 70–80% of confluence was achieved. After 24-h incubation, the cells were stained with 5 μM CellROX® Orange Reagent and Hoechst 33342 (5 μg/mL) by adding the probe to the complete media and incubating at 37 °C for 30 min. Then, the cells were washed with PBS and imaged on a Nikon Eclipse Ti inverted microscope using a 20X objective with NIS-Elements Imaging Softwere (Nikon, Tokyo, Japan).

### Determination of DNA oxidative damage

The cells were seeded into 6-well plates in concentration of 1.5 × 10^5^ cells/mL. The tested compounds were added when 70–80% of confluence was achieved. After 24-h incubation, the DNA was isolated with the Syngen DNA Mini Kit (Syngen, Poland) according to the manufacturer’s protocol. A concentration and a purity of the genomic DNA were measured using the MaestroNano Micro-Volume Spectrophotometer (Maestrogen Inc., Taiwan) and adjusted to 100 μg/mL in the TE buffer. The oxidative DNA damage was evaluated by measuring the amount of abasic sites (the so-called AP) with the DNA Damage Quantification Kit (Dojindo, Japan) according to the manufacturer’s instructions. Oxidative attacks by ROS on the deoxyribose moiety lead to the release of free bases from DNA, generating strand breaks with various sugar modifications and simple abasic sites. An aldehyde-reactive probe (ARP; N′-aminooxymethylcarbonylhydrazin-D-biotin) reacts specifically with an aldehyde group present on the open ring form of AP sites, making it possible to detect the DNA modifications that result in the formation of an aldehyde group. Biotin-avidin-specific connection and horseradish peroxidase were used for a colorimetric detection at 650 nm using PowerWave™ microplate spectrophotometer (BioTek Instruments, USA).

### DNA damage – double strand breakes (DSB)

The cells were seeded into 96-well plates in a concentration of 1.5 × 10^5^ cells/mL. The tested compounds were added when 70–80% of confluence was achieved. After 24-h incubation the genotoxicity of tested compounds in HepG2 cells was determined using the HCS DNA Damage Kit (Invitrogen, USA) according to the manufacturer’s instruction. The DNA damage was measured by a specific antibody-based detection of phosphorylated H2AX (Ser139) in the nucleus, which is induced in response to double-strand breaks (DSB) formation. The fluorescence of Alexa Fluor® 555 secondary antibody was measured using the SpectraMax i3 Multi-Mode Platform (Molecular Devices, USA).

### Lipid peroxidation (LPO)

The LPO assay is based on a malondialdehyde (MDA) and 4-hydroxyalkenals concentration (4HAE) (OxisResearch, USA). The principle underlying a lipid peroxidation assessment is based on the reaction of a chromogenic reagent N-methyl-2-phenylindole (R1) with MDA and 4HAE at 45 °C. Two molecules of R1 react with one molecule of MDA or 4HAE to form a chromophore with an absorbance maximum at 586 nm. Measuring the concentration of MDA in combination with 4HAE in methane sulfonic acid was used as an indicator of the lipid peroxidation. The assay was conducted according to the manufacturer’s instructions (OxisResearch, USA). The cells were seeded into 75 cm^2^ culture flasks in a concentration of 1.5 × 10^5^ cells/mL. Tested compounds were added when 70–80% of confluence was achieved. After 24-h incubation cells were harvested and the recommended number of 1 × 10^7^ cells was used for the analysis.

### The quantitative real-time PCR analysis (qRT-PCR)

The qRT-PCR method was used to evaluate the expression of selected genes in the HepG2 cell line. The cells were seeded into 6-well plates in the concentration of 1.5 × 10^5^ cells/mL. Tested compounds were added when 70–80% of confluence was achieved. After 24-h incubation, the cells were harvested using trypsin. The RNA was isolated from the cell line using Syngen Blood/Cell RNA Mini Kit (Syngen Biotech, Poland) and reverse transcribed with NG dART RT-PCR kit (EURx, Poland) according to the manufacturer’s instructions. The relative expression of genes encoding: hexokinase 2 and lactate dehydrogenase A (*HK2*, Hs00606086_m1; *LDHA*, Hs00855332_g1;TaqMan Gene Expression Assays, Life Technologies, USA) was determined by qRT-PCR and the ΔΔCt method using glyceraldehyde-3-phosphate dehydrogenase (*GAPDH*, Hs02758991_g1) as an endogenous control. The reaction was carried out in triplicates using the 7500 Fast Real-Time PCR System (Applied Biosystems, USA) and TaqMan Fast Universal PCR Master Mix (2x) (Applied BioSystems, USA) according to the manufacturer’s instructions. The data quality screen based on amplification curves and Ct values was performed to remove any outlier data before ΔΔCt calculations and to determine fold change in mRNA levels. The statistical analysis was performed on RQ values (RQ = 2-^ΔΔCt^).

### Statistical analysis

The results were analysed statistically in the STATISTICA vs. 12 application (StatSoft, Poland). The data were calculated as mean ± SD. To compare more than two groups, the one-way analysis of variance (ANOVA) and post hoc multiple comparisons on a basis of Tukey’s HSD test were used. All parameters were considered statistically significantly different if *p* values were less than 0.05.

## Results

### The cytotoxicity analyses

**Fig. 2:**
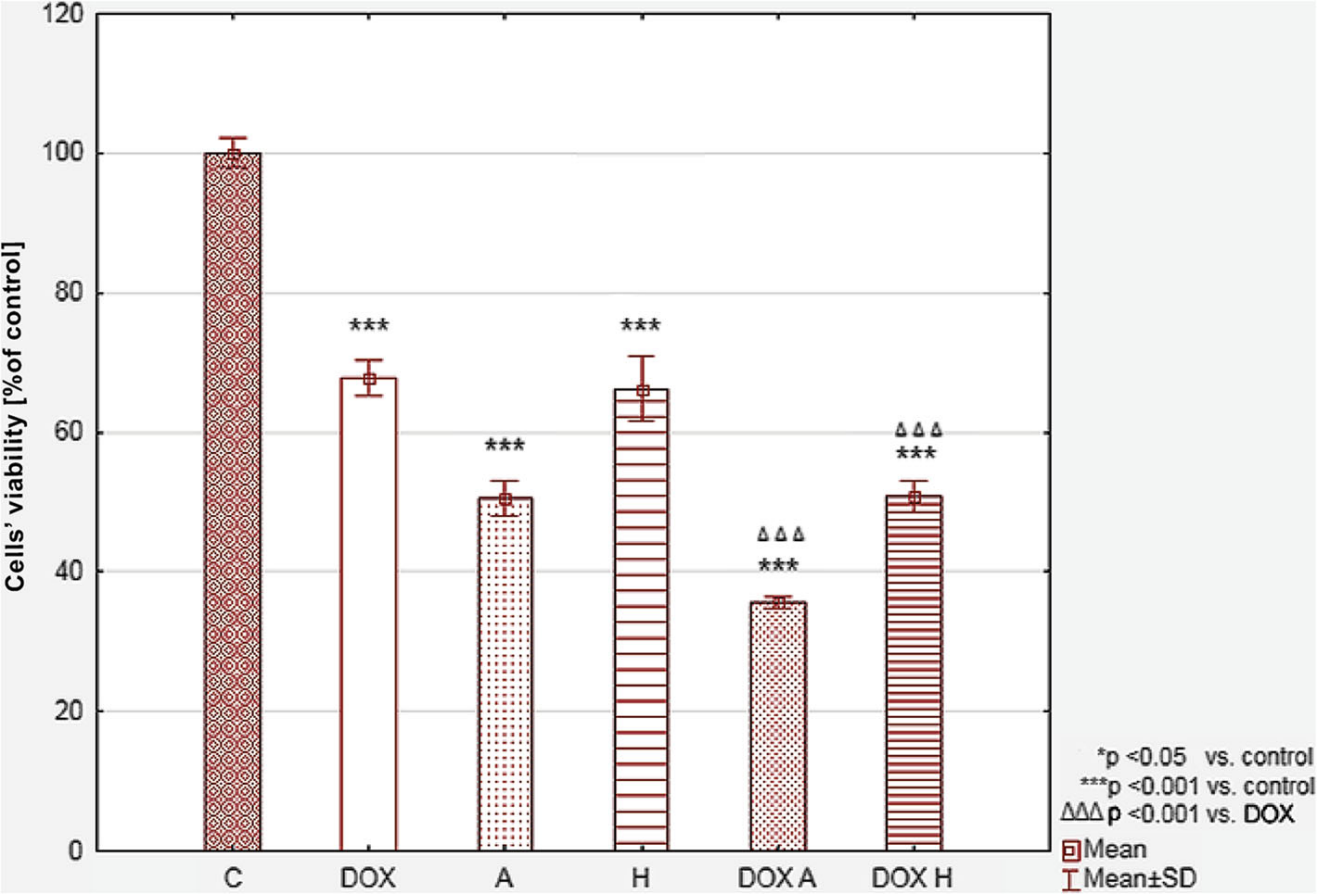
The relative HepG2 cell viability determined by MTT assay. The results were calculated as % of control cultures which were averaged to define the 100%. Values were presented as mean ± SD value of three independent experiments. To compare more than two groups, the one-way analysis of variance (ANOVA) and post hoc multiple comparisons on a basis of Tukey’s HSD test were used. DOX – 1 μM doxorubicin, A – 100 μM apigenin, H – 200 μM hesperidin, DOX A – 1 μM doxorubicin and 100 μM apigenin, DOX H – 1 μM doxorubicin and 200 μM hesperidin

The MTT assay revealed that 1 μM DOX has moderate impact on HepG2 cell’s viability. In this case the cell’s viability was lowered to 67.77 ± 2.43% (Table 1, Fig. 2). To sensitize the cells on this chemotherapeutic, the combination of DOX and following flavonoids was applied: apigenin, cosmosiin, rhoifolin, baicalein, baicalin, hesperetin and hesperidin. Only apigenin (100 μM) and hesperidin (200 μM) managed to sensitize the cells on DOX (viability 35.62 ± 0.73 and 50.85 ± 2.28%, respectively). Furthermore, both flavonoids in above concentrations caused cytotoxicity in HepG2 cells (viability 50.55 ± 2.60 and 66.55 ± 3.87%, respectively).

**Table 1:** HepG2 cells’ viability after treatment with doxorubicin (DOX), apigenin (A), hesperidin (H), hesperetin (HAGL), baicalin (B), baicalein (BAGL), cosmosiin (C), rhoifolin (R) and tested compounds treated simultaneously with doxorubicin. Data are presented as a mean ± SD % of a control

Concentration [μM]	Control	DOX	A			H			HAGL			B			BAGL			C			R		
		1	200	100	50	200	100	50	200	100	50	200	100	50	200	100	50	200	100	50	200	100	50
	Mean ± SD %																						
	100.04 ± 2.99																						
Compound alone		67.77 ± 2.44	45.66 ± 3.44*	50.55 ± 2.60*	67.64 ± 3.85*	66.55 ± 3.87*	75.85 ± 3.54*	82.65 ± 3.32*	81.64 ± 1.73*	97.24 ± 2.50	99.67 ± 3.45	81.50 ± 2.60*	87.74 ± 4.41*	90.52 ± 3.35*	74.04 ± 3.21*	77.82 ± 7.44*	81.73 ± 5.34*	100.81 ± 4.07	101.05 ± 2.13	99.87 ± 6.12	98.35 ± 7.08	99.13 ± 1.91	106.17 ± 9.50
Compound + DOX			32.75 ± 1.15♦	35.62 ± 0.73♦	52.31 ± 0.47♦	50.85 ± 2.28♦	62,23 ± 5.32♦	63.88 ± 3.55♦	78.96 ± 2.04	87.53 ± 3.97♦	86.55 ± 5.38♦	79.84 ± 2.65	80.79 ± 5.26	80.99 ± 3.46♦	68.79 ± 1.59	69.71 ± 3.20	69,11 ± 5.54	82.23 ± 5.08♦	76.77 ± 1.50♦	79.64 ± 2.18♦	73.02 ± 2.15♦	80.26 ± 1.75♦	87.03 ± 3.43♦

* *p* ≤ 0.05 vs Control

♦ *p* ≤ 0.05 vs DOX

### Cells’ morphology

**Fig. 3:**
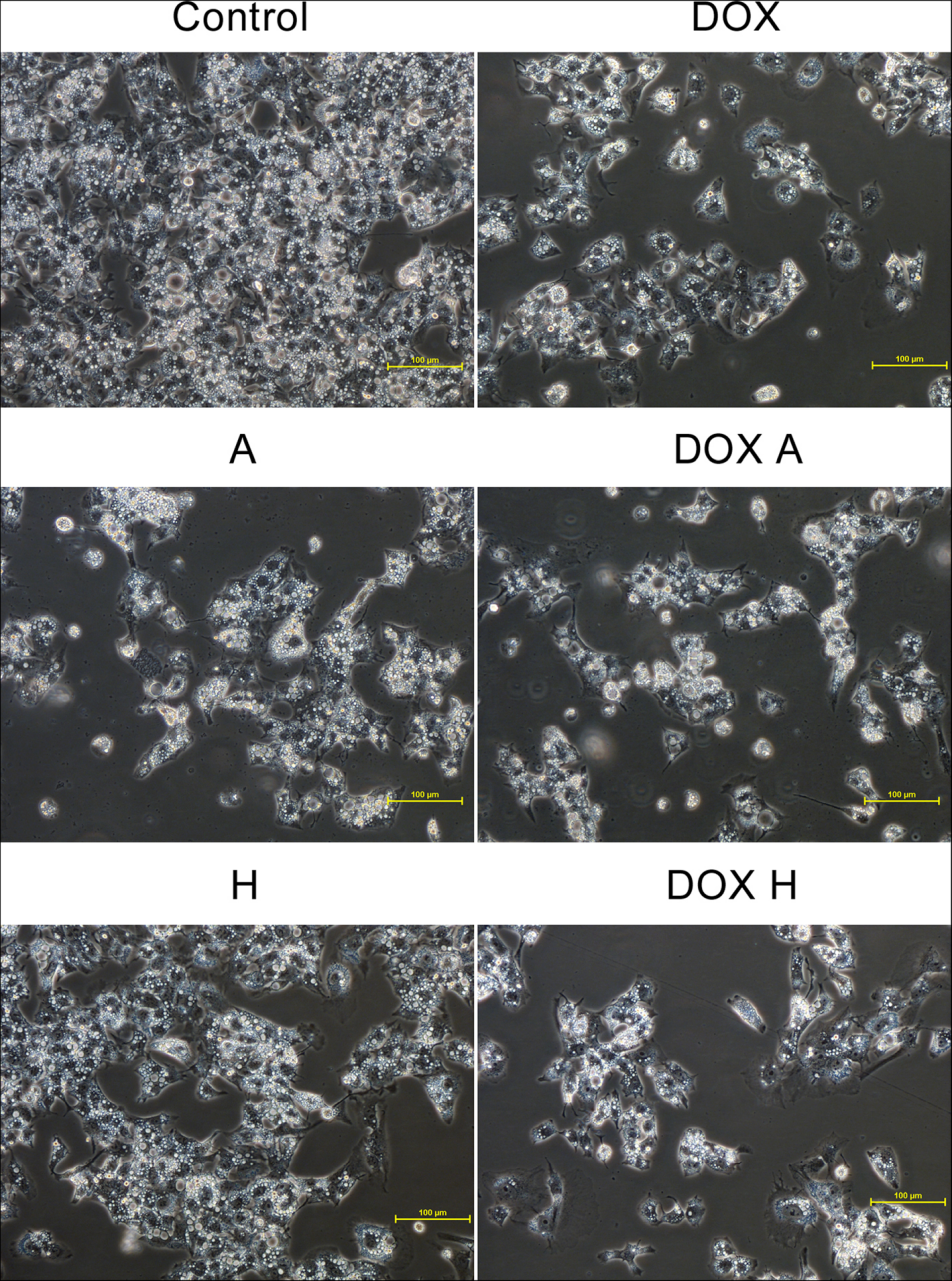
Morphological changes of HepG2 cells. The control cells showed a normal morphology, they were closely arranged and well adherent in large numbers. After treatment with tested compounds (DOX, apigenin, hesperidin or combined) the number of normal cells was significantly reduced, the cells became round and had poor adherence, especially the DOX A and DOX H treated cultures. The results present one representative experiment of three independently performed that showed similar patterns. HepG2 cell morphology was analysed under a phase-contrast microscope Nikon Eclipse Ti, magnification x200, scale bar = 100 μm. C – control, DOX – 1 μM doxorubicin, A – 100 μM apigenin, H – 200 μM hesperidin, DOX A – 1 μM doxorubicin and 100 μM apigenin, DOX H – 1 μM doxorubicin and 200 μM hesperidin

The effects of 100 μM apigenin and 200 μM hesperidin in the presence or absence of 1 μM DOX on HepG2 cell morphology were analysed under a phase-contrast microscope Nikon Eclipse Ti. The control cells showed a normal morphology, they were closely arranged and well adherent in large numbers. After treatment with tested compounds (DOX, apigenin, hesperidin or combined) the number of normal cells was significantly reduced, the cells became round and had poor adherence, especially in cultures simultaneously treated with combination of DOX and flavonoid. The smallest intensity of observed changes was visible in cell cultures treated with hesperidin alone (Fig. 3).

### ROS level detection

**Fig. 4:**
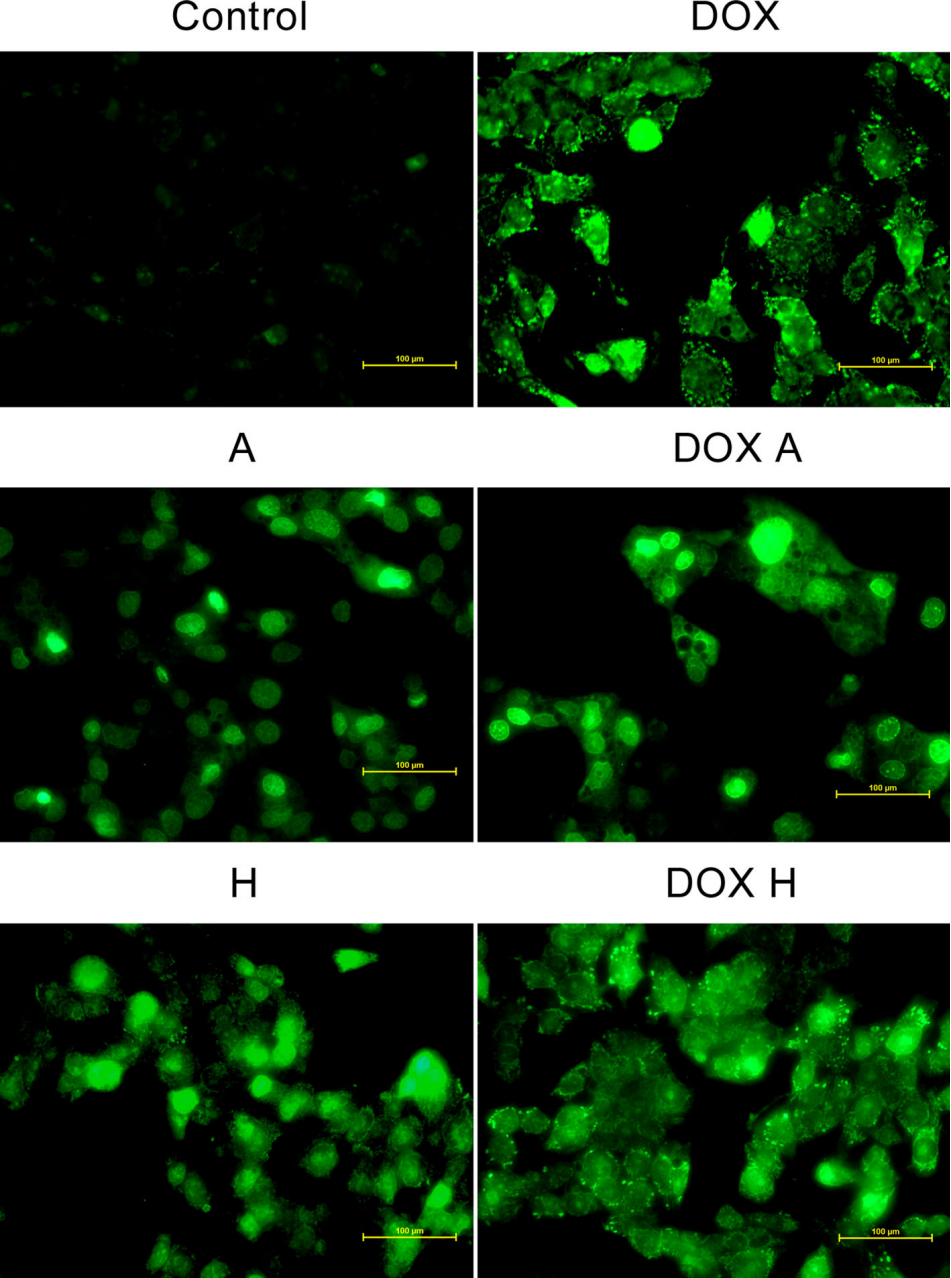
The detection of ROS generation using CellROX Green Reagent. In cells treated with DOX there was a high fluorescent signal deriving from mitochondria. In the case of A cultures the signal came from the nuclei. In DOX A, the signal came only from the nuclei. H showed oxidative signal in nuclei, however DOX and H combination – in both nuclei and mitochondria. The results present one representative experiment of three independently performed that showed similar patterns. HepG2 cells morphology was analysed under a phase-contrast microscope Nikon Eclipse Ti, magnification x300, scale bar = 100 μm. C – control, DOX – 1 μM doxorubicin, A – 100 μM apigenin, H – 200 μM hesperidin, DOX A – 1 μM doxorubicin and 100 μM apigenin, DOX H – 1 μM doxorubicin and 200 μM hesperidin

Upon oxidation, CellROX Green reagent binds to DNA and thus its signal is localized primarily in the nucleus and mitochondria. After labelling the cells with the CellROX Green Reagent, a high fluorescent signal deriving from mitochondria was observed in cells treated with DOX. In the case of apigenin the signal came from the nuclei. The combined treatment showed that the signal came only from the nuclei. Hesperidin similarly to apigenin generated the oxidative stress signal in the nuclei, however hesperidin and DOX combined – in both nuclei and mitochondria (Fig. 4) .

### Determination of DNA oxidative damage

**Fig. 5:**
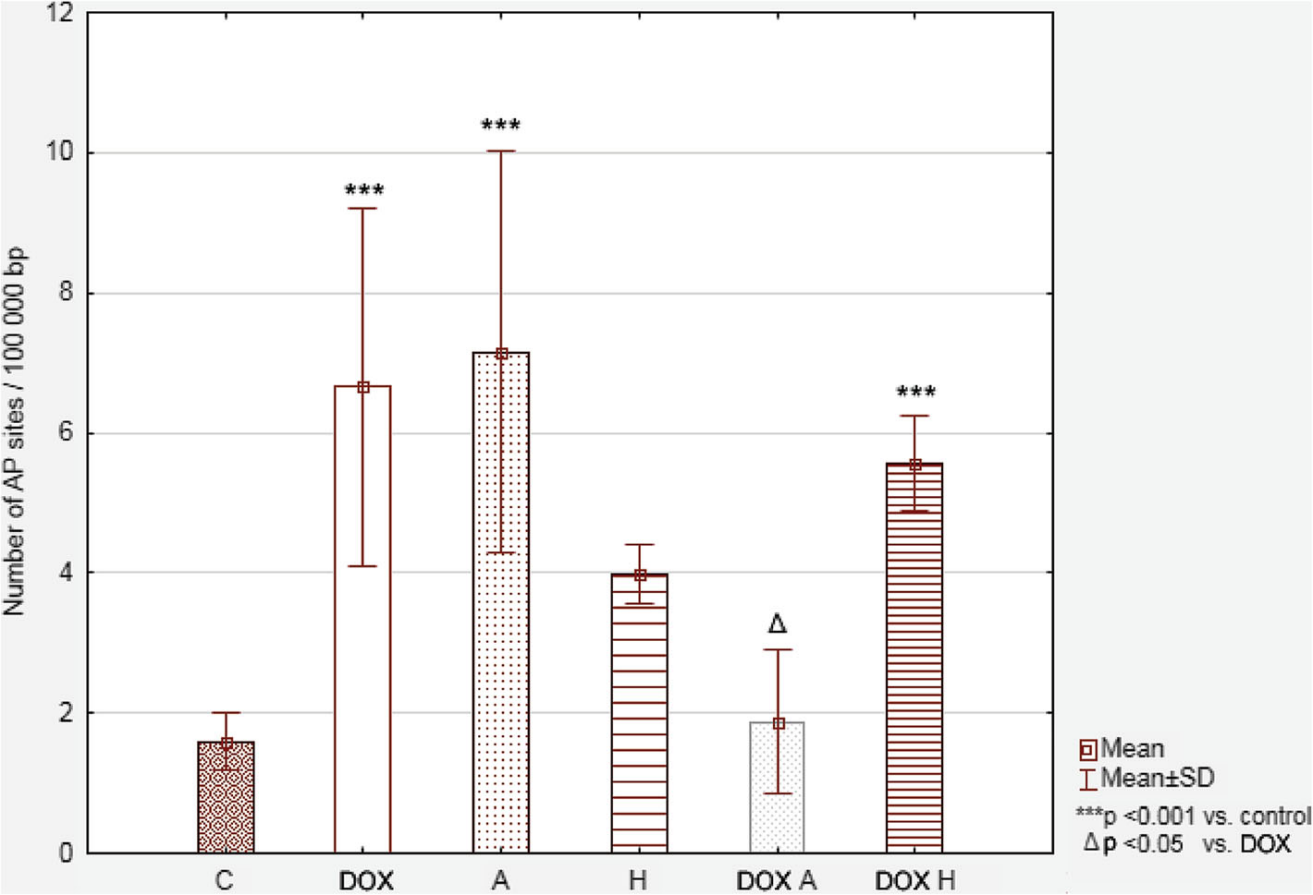
AP sites’ number per 100,000 bp in HepG2 cell line. Values were presented as mean ± SD. To compare more than two groups, the one-way analysis of variance (ANOVA) and post hoc multiple comparisons on a basis of Tukey’s HSD test were used. C – control, DOX – 1 μM doxorubicin, A – 100 μM apigenin, H – 200 μM hesperidin, DOX A – 1 μM doxorubicin and 100 μM apigenin, DOX H – 1 μM doxorubicin and 200 μM hesperidin

The assessment of oxidative DNA damage showed a significant increase of the AP sites accumulation in the DNA isolated from the HepG2 cells treated with DOX (6.65 ± 2.56 AP sites/100 000 bp) as well as apigenin (7.15 ± 2.87 AP sites/100 000 bp) and hesperidin (3.98 ± 0.42 AP sites/100 000 bp) in comparison to the control culture (1.59 ± 0.41 AP sites/100 000 bp, see Fig. 5). After combining DOX with apigenin, we observed drop in the level of AP-sites (1.88 ± 1.02/100 000 bp). However, there was no significant change after treatment with both DOX and hesperidin (5.55 ± 0.67/100 000 bp) in comparison to DOX alone.

### DNA damage – double strand breaks (DSB)

**Fig. 6:**
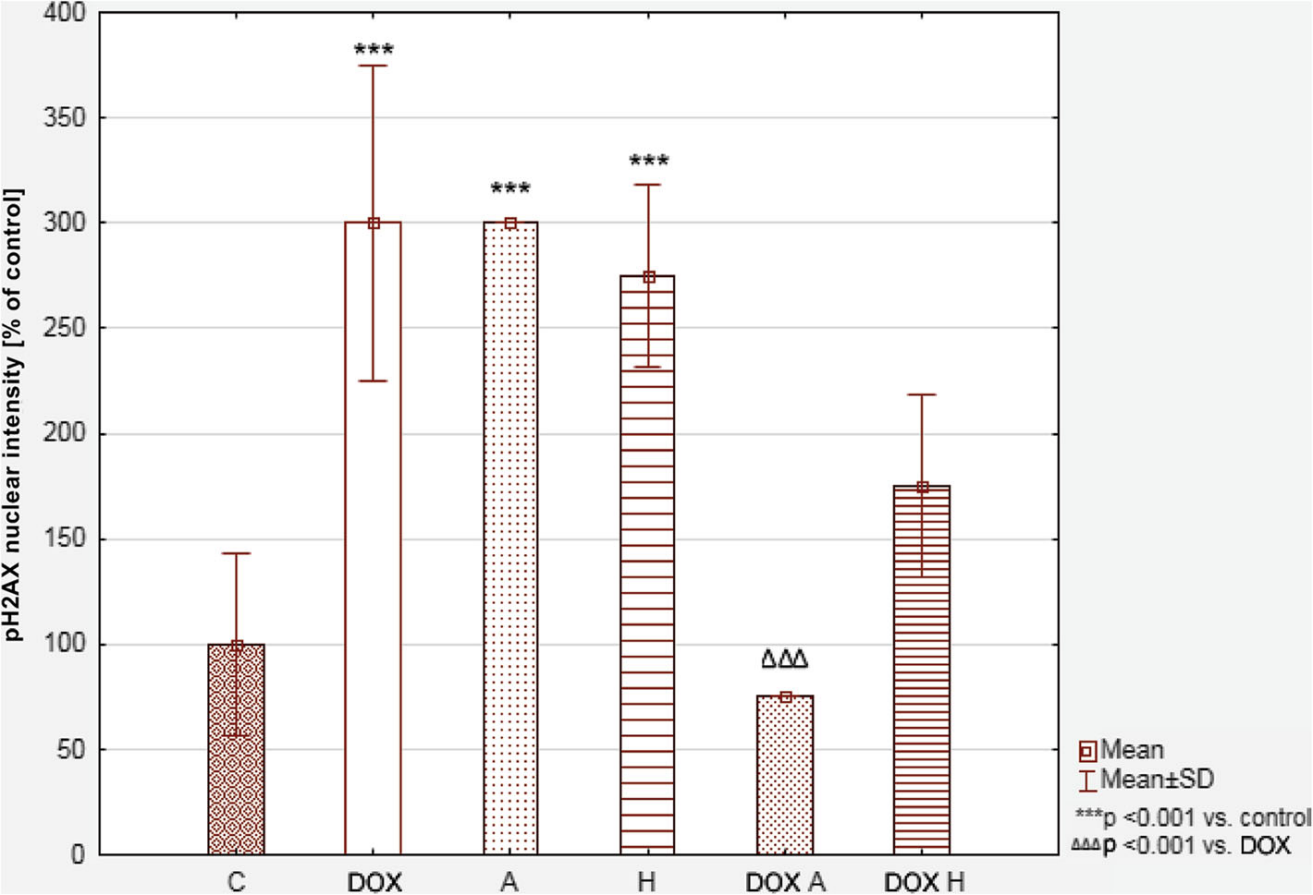
The content of DSB in tested cell’s DNA (based on phosphorylated H2AX level) presented as a % of a control. Values were presented as mean ± SD. To compare more than two groups, the one-way analysis of variance (ANOVA) and post hoc multiple comparisons on a basis of Tukey’s HSD test were used. C – control, DOX – 1 μM doxorubicin, A – 100 μM apigenin, H – 200 μM hesperidin, DOX A – 1 μM doxorubicin and 100 μM apigenin, DOX H – 1 μM doxorubicin and 200 μM hesperidin

The DSB were measured by specific antibody-based detection of phosphorylated H2AX in the nucleus. The H2AX level was much higher after treating the cells with compounds alone than in the control cells (300 ± 75; 300; 275 ± 43.30% for DOX, apigenin and hesperidin, respectively; see Fig. 6). After combining DOX with apigenin, a significant decrease of phosphorylated H2AX in the nucleus was observed. In this case its level constituted 75% of the control. Combining DOX with hesperidin had no significant impact on the H2AX phosphorylation when compared to DOX alone.

### Lipid peroxidation

**Fig. 7:**
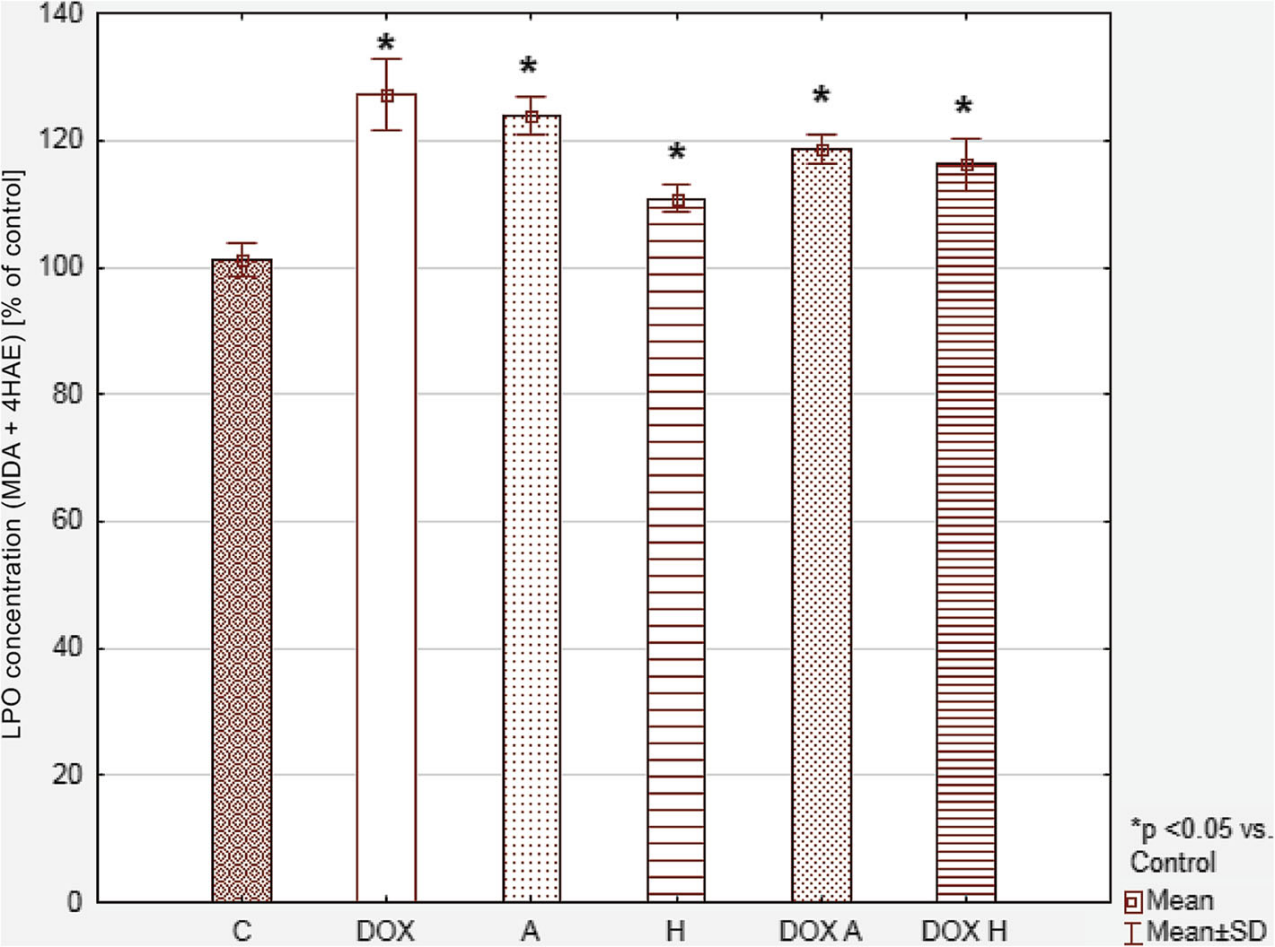
Lipid peroxidation level in HepG2 cells on the basis of MDA and 4-HAE concentration, presented as a % of a control. Values were presented as mean ± SD. To compare more than two groups, the one-way analysis of variance (ANOVA) and post hoc multiple comparisons on a basis of Tukey’s HSD test were used. C – control, DOX – 1 μM doxorubicin, A – 100 μM apigenin, H – 200 μM hesperidin, DOX A – 1 μM doxorubicin and 100 μM apigenin, DOX H – 1 μM doxorubicin and 200 μM hesperidin

The MDA + 4HAE levels were higher in all tested samples as compared to the control, but they did not significantly differ from each other (Fig. 7).

### The quantitative real-time PCR analysis (qRT-PCR)

**Fig. 8:**
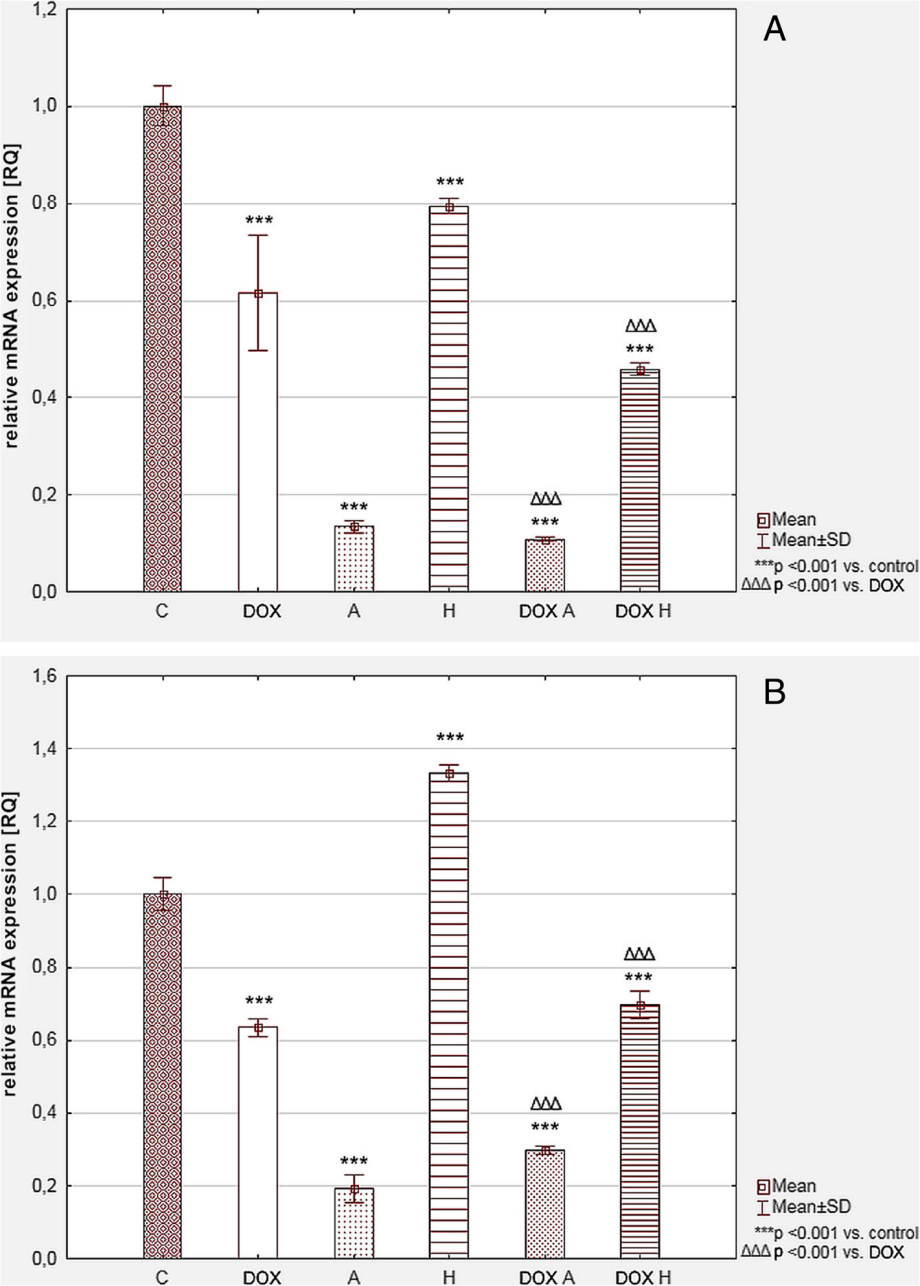
Relative mRNA expression level of *HK2* (**a**) and *LDH-A* (**b**) in tested cells. *GAPDH* was used as a reference gene. The results were calculated as RQ values and presented as mean ± SD. To compare more than two groups, the one-way analysis of variance (ANOVA) and post hoc multiple comparisons on a basis of Tukey’s HSD test were used. C – control, DOX – 1 μM doxorubicin, A – 100 μM apigenin, H – 200 μM hesperidin, DOX A – 1 μM doxorubicin and 100 μM apigenin, DOX H – 1 μM doxorubicin and 200 μM hesperidin

The results showed that DOX alone decreased *HK2* and *LDHA* expression – RQ = 0.615 ± 0.132 and 0.635 ± 0.026 respectively (see Fig. 8a, b). After apigenin treatment both *HK2* and *LDHA* expression were about 5-fold lower than in the control (0.135 ± 0.013 and 0.191 ± 0.042). Combining both compounds also inhibited these enzymes’ gene expression to the level of RQ = 0.108 ± 0.004 for *HK2* and RQ = 0.298 ± 0.013 for *LDHA*. In DOX and hesperidin combination no hesperidin influence was observed (RQ = 0.458 ± 0.015 and RQ = 0.697 ± 0.043). However, hesperidin alone decreased *HK2* and increased *LDHA* expressions (RQ = 0.795 ± 0.016 and RQ = 1.332 ± 0.024, respectively).

## Discussion

The HepG2 cell line used for the study is being commonly used as a model of the hepatocellular carcinoma (HCC). In the clinic, the maximum DOX concentration in the blood reaches 10 μM. However, 1 μM is the most commonly used concentration. In the conducted studies, 1 μM of DOX showed a significant effect on HepG2 cells, reducing the cells’ viability by approximately 30%. Poor response to DOX therapy is also observed in systemic chemotherapy in patients with advanced HCC. The resistance mechanism is usually complex and multidirectional. It is postulated, among others, participation in the mechanism of multidrug resistance [19, 20] and changes in the metabolic phenotype - Warburg effect. The Warburg effect is based on the activation of glycolysis in cancer cells even though the cell’s oxygenation is normal [8, 9]. Usually, glycolysis is activated during oxygen deficiency and is observed during the growth of solid tumours [21]. Both hypoxia and Warburg effect, are associated with an increased glucose uptake by a cell what occurs in about 80% [21] of all known cancers and is being used with great success in PET diagnostics [11, 21]. For this reason, the strategy of inhibiting glycolysis in the fight with cancer seems justified. A number of studies have already shown that inhibiting of glycolysis pathway inhibits the proliferation, kills the cancer cells [22–27] or makes the cancer cells more sensitive to chemotherapeutic agents [17]. Hexokinase, e.g., catalyses the first and rate-limiting reaction in glycolysis. Several studies demonstrate that hexokinase, particularly its second isoform (HK2), plays a critical role in initiating and maintaining the high glucose catabolic rates of rapidly growing tumours. Most immortalized and malignant cells display increased expression of HK2, which might contribute to elevated glycolysis [28–30]. At the genetic level, certain tumour cells exhibit increased gene copy number of HK2.

Lactate dehydrogenase is a tetramer of A and B subunits, encoded by two separate genes. This enzyme catalyses the conversion of pyruvate to lactate coupled with an oxidation of NADH to NAD^+^, which is essential for the glycolytic pathway. Interestingly, *LDHA* gene is controlled by hypoxia inducible factor (HIF-1α), whereas *LDHB* gene is not regulated by low oxygen concentration. In addition to its essential role in glucose metabolism, *LDHA* isoform has been identified as a single-stranded-DNA-binding protein [31, 32]. Recent biochemical studies suggest that *LDHA* and *LDHB* are components of a cell-cycle-dependent transcriptional coactivator [33]. These unexpected observations indicate that a fraction of LDH might participate in DNA replication and RNA transcription. Recently, studies have shown a positive effect of *LDHA* inhibition on the radiosensitivity of gliblastoma cells [34].

Our preliminary studies have shown that apigenin inhibited the expression of the *LDHA* and *HK2* genes very strongly while the hesperidin inhibited the expression of *HK2* but intensified the expression of *LDHA*. Therefore, for the current research, we chose apigenin in the belief that reduced *LDHA* expression is likely to lead to a decrease in the concentration and activity of the LDHA and HK2 protein. DOX alone also inhibited the expression of both genes but to a much lesser extent than apigenin. In the cultures of cells incubated together with DOX and apigenin, a significant decrease in the expression of both genes was found, but to the level observed for apigenin itself, indicating that the effect in the DOX + apigenin cultures is mainly caused by apigenin. MTT assay and examination on the microscope showed a clear synergism of the cytotoxic effects of DOX and apigenin. Considering the profile of changes in *LDHA* and *HK2* mRNA expression described above, it can be concluded that this synergism is accompanied by lowered mRNA level of glycolytic genes, however we cannot state that glycolytic disturbances are pivotal for this phenomenon.

Since oxidative stress and the interaction with DNA play a significant role in the mechanism of action of DOX, the parameters of oxidative stress and DSB have been studied. In each of studied culture (i.e. DOX, apigenin, DOX + apigenin) the lipid peroxidation was increased, but no interaction was demonstrated between DOX and apigenin. However, with respect to the DNA, both DOX and apigenin were found to significantly increase oxidative damage to DNA and DSB.

There are few reports about the DNA damage caused by apigenin [35, 36]. Arango et al. stated that apigenin induces DNA damage through down-regulation of the genes involved in a cell cycle control and DNA repair. Vrhovac Madunić et al., who treated human breast cancer cells with apigenin, observed genotoxic effect attributed to an oxidative stress. Hesperidin was also reported to damage DNA in the skin cancer cell line [37]. It is also known that flavonoids can intercalate DNA duplex as well as act as topoisomerase poison [38, 39]. The exact mechanism of DNA damage (oxidative and DSB) by flavonoids must be elucidated. It is an interesting issue, because flavonoids are Janus-faced compounds – they are known for their protective activities, especially in the context of an oxidative stress. On the other hand, they can be toxic for cancer cells and damage DNA as it has been revealed in the present study. Other tested compounds did not reveal synergistic toxic effect. What is more, they showed protective activity towards cancer cells in a presence of DOX. Protective activities are mainly attributed to antioxidant properties of flavonoids that result from radical scavenging activity and the enzyme functioning interaction [40].

Interestingly, the combined administration of both agents – DOX and apigenin leads to complete normalization of the DNA oxidative damage and DSB, which indicates a rather rare type of interaction. The research showed that the synergism of the cytotoxic effects of DOX and apigenin are not dependent on DNA damage. It can even be said that the oxidative damage of DNA and DSB caused by DOX are abolished by apigenin. For this reason, another test was carried out to extend the conclusion in the field of oxidative stress. It has been shown that incubation of cells with DOX leads to an increase ROS level outside the cell nucleus, probably in the mitochondria. Apigenin alone induced ROS in the cell nucleus, which is consistent with the DNA oxidative damage test, but the combined administration of DOX with apigenin gives a similar effect as for apigenin itself – ROS presence in the cell nucleus, in the absence of extranuclear (mitochondrial) signal. It condemns it to suppress the mitochondrial ROS production (induced by DOX) as a result of apigenin action. Doxorubicin is known for its mitochondrial accumulation and generation of ROS in several ways, however this mechanism is mainly contributed to pathogenesis of DOX cardiotoxicity rather than anticancer action [41]. Regardless of that, the obtained results suggest that apigenin can counteract the transport of DOX into the mitochondria through an unknown mechanism. This phenomenon was not observed in case of hesperidin.

It is difficult to explain, why during the simultaneous action of DOX and apigenin, the presence of ROS in the cell nucleus is observed, while DNA damage is minimized. There is a report which may explain this interesting phenomenon. Rusak et al. studied the influence of selected flavonoids on lymphocyte’s DNA [42]. They observed that flavonoids can cause DNA damage as well as act in a protective way in the presence of an oxidative stress factor. In addition, it has been known that both DOX and flavonoids intercalate to the DNA [39]. Thus, one may suspect that there may be a competition between them.

Flavonoids demonstrate a number of biological properties. The dependence of these properties and the flavonoid’s structure is being widely studied. Anticancer properties are attributed to C2 = C3 double bond, pattern of hydroxylation (6-OH and 5,7-diOH in A ring and 3-OH in C-ring) [43], however further examination is needed. In present study, there is no simple structure-activity correlation. Only apigenin and hesperidin influenced DOX toxicity in tested cells. This two compounds differ in both glycosylation and hydroxylation pattern. What is more, they affected DOX cytotoxicity in a different way. It was initially supposed that the biological activity of flavonoids would be related to their antioxidant properties. However, available evidence from cell culture experiments suggested that many biological effects of flavonoids are related to their ability to modulate enzymatic activity, gene expression or DNA intercalation and even prooxidative properties [40, 43, 44]. The present study confirmed this observation. This multidirectional activity causes that the effect of their action depends on many other than just the structure factors such as concentration, other compounds presence, type of cells.

## Conclusions

In conclusion, apigenin application intensifies the antitumor effect of DOX, which may be an interesting therapeutic proposition in both systemic therapy supplementation and chemoembolization of hepatocellular carcinoma. The synergism of anticancer activity is not related to oxidative stress but is accompanied by inhibition of glycolytic genes expression. To explain this synergy mechanism, further research is needed, above all on the effect of apigenin on topoisomerase inhibition by DOX.

## Abbreviations

A: Apigenin
DOX: Doxorubicin
DSB: Double strand breaks
GAPDH: Glyceraldehyde 3-phosphate dehydrogenase
HCC: Hepatocellular carcinoma
HK2: Hexokinase 2
LDHA: Lactate dehydrogenase A
MTT: 3-(4,5-dimethylthiazol-2-yl)-2,5-diphenyltetrazolium bromide
ROS: Reactive oxygen species
